# A double-edged sword: e-cigarettes, and other electronic nicotine delivery systems (ENDS): reply

**DOI:** 10.1007/s11739-019-02228-8

**Published:** 2019-11-14

**Authors:** Riccardo Polosa, Konstantinos Farsalinos, Domenico Prisco

**Affiliations:** 1 Centro per la Prevenzione e Cura del Tabagismo (CPCT), Azienda Ospedaliero-Universitaria “Policlinico-V. Emanuele”, Università of Catania, Catania, Italy; 2 Dipartimento di Medicina Clinica e Sperimentale (MEDCLIN), University of Catania, Catania, Italy; 3 Center of Excellence for the Acceleration of HArm Reduction (CoEHAR), University of Catania, Catania, Italy; 4 Department of Cardiology, Onassis Cardiac Surgery Center, Kallithea, Greece; 5 Department of Pharmacy, University of Patras, Patras, Greece; 6 National School of Public Health, Athina, Greece; 7 Department of Experimental and Clinical Medicine, University of Florence, Florence, Italy; 8 Interdisciplinary Internal Medicine, Careggi University Hospital, Florence, Italy

Dear Editor,

Disagreement in a scientific debate is healthy.

Although we commend the intent of Lal et al. [1] to further elaborate on the impact of e-cigarettes and other electronic nicotine delivery systems (ENDS) on human health, we disagree on some of these authors’ assessment of the literature. And we certainly disagree with their statement that e-cigarettes are “becoming a nuisance for the society” when it is evident that these tar-free-emitting technologies are displacing cigarette smoking globally.

While several studies have found that e-cigarette use at baseline predicts smoking at a later period, there is convincing evidence that there is a bidirectional association, with smoking at baseline predicting follow-up e-cigarette use too (Table 1). In fact, it is more plausible for these findings to be explained by the common liability model, rather than the gateway model [2]. Further indications for the absence of gateway to smoking effects for e-cigarettes come from examining the smoking rates among US youth over time. Reductions of > 60% and 50% in middle school and high school students’ rates of past 30 days smoking have been reported from 2011 to 2018, the period when e-cigarettes became very popular (Table 1). US youth now have the lowest smoking rates that have historically been recorded, with an accelerated rate of decline compared to previous years. Combined with the minimal rates of frequent e-cigarette use among never-smoking youth, it is likely that e-cigarettes have distracted US youth from smoking rather than recruiting more smokers (Table 1).

**Table 1:** Debunking key concerns about e-cigarettes

Concern	Key debunking references
Gateway effect	East K, Hitchman SC, Bakolis I, Williams S, Cheeseman H, Arnott D, McNeill A. The Association Between Smoking and Electronic Cigarette Use in a Cohort of Young People. J Adolesc Health. 2018;62:539–547. 10.1016/j.jadohealth.2017.11.301. Leventhal AM, Strong DR, Kirkpatrick MG, Unger JB, Sussman S, Riggs NR, Stone MD, Khoddam R, Samet JM, Audrain-McGovern J. Association of electronic cigarette use with initiation of combustible tobacco product smoking in early adolescence. JAMA. 2015;314:700–707. Centers for Disease Control and Prevention. Youth and Tobacco Use. 2019. Available at: https://www.cdc.gov/tobacco/data_statistics/fact_sheets/youth_data/tobacco_use/index.htm Farsalinos K, Tomaselli V, Polosa R. Frequency of Use and Smoking Status of U.S. Adolescent E-Cigarette Users in 2015. Am J Prev Med. 2018 Jun;54(6):814–820.
Flavor appeal	Buu A, Hu YH, Piper ME, Lin HC. The association between e-cigarette use characteristics and combustible cigarette consumption and dependence symptoms: Results from a national longitudinal study. Addict Behav. 2018 Sep;84:69–74. 10.1016/j.addbeh.2018.03.035. Epub 2018 Apr 3. Farsalinos KE, Romagna G, Tsiapras D, Kyrzopoulos S, Spyrou A, Voudris V. Impact of flavour variability on electronic cigarette use experience: an internet survey. Int J Environ Res Public Health. 2013 Dec 17;10(12):7272-82. 10.3390/ijerph10127272.
Cancer risk	Farsalinos KE, Gillman G. Carbonyl Emissions in E-cigarette Aerosol: A Systematic Review and Methodological Considerations. Front Physiol. 2018 Jan 11;8:1119. 10.3389/fphys.2017.01119. Shahab L, Goniewicz ML, Blount BC, Brown J, McNeill A, Alwis KU, Feng J, Wang L, West R. Nicotine, Carcinogen, and Toxin Exposure in Long-Term E-Cigarette and Nicotine Replacement Therapy Users: A Cross-sectional Study. Ann Intern Med. 2017 Mar 21;166(6):390–400. 10.7326/m16-1107. Stephens WE. Comparing the cancer potencies of emissions from vapourised nicotine products including e-cigarettes with those of tobacco smoke. Tob Control. 2017 Aug 4. pii: tobacco control-2017-053808. 10.1136/tobaccocontrol-2017-053808. Scungio M, Stabile L, Buonanno G. Measurements of electronic cigarette-generated particles for the evaluation of lung cancer risk of active and passive users. Journal of Aerosol Science 2018; 115: 1–11.
COPD/asthma risk	Polosa R, Morjaria JB, Prosperini U, Russo C, Pennisi A, Puleo R, Caruso M, Caponnetto P. Health effects in COPD smokers who switch to electronic cigarettes: a retrospective-prospective 3-year follow-up. Int J Chron Obstruct Pulmon Dis. 2018 Aug 22;13:2533–2542. 10.2147/copd.s161138. Polosa R, Morjaria JB, Caponnetto P, Caruso M, Campagna D, Amaradio MD, Ciampi G, Russo C, Fisichella A. Persisting long term benefits of smoking abstinence and reduction in asthmatic smokers who have switched to electronic cigarettes. Discov Med. 2016 Feb; 21(114):99–108.

While flavors could appeal to youth, they are particularly important for adult former smokers who are using e-cigarettes as smoking substitutes, and could possibly contribute to successfully quitting and preventing relapse (Table 1). Of course, it is not desirable for youth (as well as never-smoking adults) to initiate e-cigarette use, and continuous monitoring of use according to smoking status is warranted. In any case, it is important to consider both potential benefits and harms of any regulatory restrictions in different population subgroups, and estimate the overall public health impact before being implemented.

The authors are also downplaying the much reduced malignancy risk of tar-free technologies compared to combustible cigarettes by citing in vitro studies that have been largely dismissed because of abnormal exposure protocols that do not replicate normal condition of use and lack of appropriate experimental controls [3]. While not risk free, there is no doubt that ECs are by far less harmful than smoking with lower toxin emissions and lower toxin exposure as evaluated by measuring biomarkers (Table 1). As noted in our recent Editorial [4], direct evidence for the reduction in lung cancer risk by suppressing tar exposure is not possible due to the substantial latency period of this disease. However, estimation of lifetime cancer risks—by modeling the cancer potencies of e-cigarette and ENDS emission aerosols using published chemical analyses of emissions and their associated inhalation unit risks—showed that the cancer risk for vapers is several orders of magnitude lower compared to smoking (Table 1), a clear indication of the tremendous harm reduction potential of e-cigarettes. The same logic applies to other smoking-related and smoking-exacerbated diseases such as COPD and asthma, in which regular long-term EC use has been shown to improve (not to worsen) subjective as well as objective respiratory outcomes (Table 1). Of note, in a 3.5-year follow-up of a small cohort of never-smoking daily EC users, no deterioration in spirometric indices, development of respiratory symptoms, changes in markers of lung inflammation nor signs of early lung damage on HRCT were noted [5]. Moreover, the recent outbreak of severe acute respiratory illnesses among several hundred US young adults and teens is NOT linked to commercial nicotine vaping products. Both the FDA (Food and Drug Administration) and the CDC (US Centers for Disease Control and Prevention) now acknowledge that the outbreak is caused by the consumption of some illegal, black market cannabis liquids containing dangerous adulterants [6, 7].

We certainly do not disagree with Lal et al. [1] that improved regulation of these new technologies is needed. In particular, enhanced monitoring of e-cigarette refills should be in place, to prevent reckless alteration and to promote quality/safety control mechanisms. We have long supported and endorsed standards through the work of Technical Committee of the European Standardization body for e-cigarettes and e-liquids (CEN TC437) to ensure that manufacturers have quality control systems in place to prevent these problems and to safeguard consumers’ health from marketing of non-approved vaping products. Nonetheless, regulation should be sensible, balanced, pragmatic, risk proportionate and evidence based. Overrestricting use of tobacco harm reduction products not only protects none but it also deprives smokers from the chance to improve their health. It also forces any youth who may unfortunately want to initiate an inhalational habit to have one option: combustible tobacco cigarettes.

We should not lose sight of the potential benefits of ECs compared to cigarettes as a lot of people still smoke conventional cigarettes and this will be a public health issue for a number of years to come.
